# Bis-(salicylaldehyde-benzhydrylimino)nickel complexes with different electron groups: crystal structure and their catalytic properties toward (co)polymerization of norbornene and 1-hexene†

**DOI:** 10.1039/c8ra06561f

**Published:** 2018-10-25

**Authors:** Xiaohui He, Guangshui Tu, Feng Zhang, Shengmei Huang, Changwen Cheng, Chuanyi Zhu, Yapeng Duan, Suli Wang, Defu Chen

**Affiliations:** School of Materials Science and Engineering, Nanchang University 999 Xuefu Avenue Nanchang 330031 China hexiaohui@ncu.edu.cn; School of Civil Engineering and Architecture, Nanchang University 999 Xuefu Avenue Nanchang 330031 China; School of Materials Science and Engineering, Nanchang Hangkong University Nanchang 330063 China

## Abstract

Eight bis-(salicylaldehyde-benzhydrylimino)nickel complexes with different electron groups (Ni1–Ni8), Ni{(3-R_1_)(5-R_2_)C_6_H_2_(O)CHNCH(C_6_H_5_)_2_}_2_, (R_1_ = H, R_2_ = H, Ni1; R_1_ = H, R_2_ = CH_3_, Ni2; R_1_ = H, R_2_ = OCH_3_, Ni3; R_1_ = H, R_2_ = Br, Ni4; R_1_ = CH_3_, R_2_ = H, Ni5; R_1_ = OCH_3_, R_2_ = H, Ni6; R_1_ = Br, R_2_ = Br, Ni7; R_1_ = Cl, R_2_ = Cl, Ni8), were synthesized and their crystal structures were characterized using single crystal X-ray diffraction. The results revealed that Ni1–Ni6 belong to the monoclinic system (space group *P*2(1)/*n*), Ni7 belongs to the monoclinic system (space group *C*2/*c*) and Ni8 belongs to the triclinic system (space group *P*1̄). All nickel complexes exhibited high activities (0.46–2.07 × 10^6^ g_polymer_ mol_Ni_^−1^ h^−1^) toward norbornene homopolymerization, and a strong electron-withdrawing group on the salicylaldimino aromatic ring can enhance the catalytic activity and favor polymerization. Ni1 and Ni2 exhibited high activities (0.55–2.40 × 10^5^ g_polymer_ mol_Ni_^−1^ h^−1^) toward copolymerization of norbornene and 1-hexene in the presence of B(C_6_F_5_)_3_. The 1-hexene content in the copolymers could be controlled up to 7.98–12.50% by varying the comonomer feed ratio of 1-hexene from 10 to 50%. It is observed that when the 5-position of the salicylaldimino aromatic ring has a substituent (–CH_3_), the 1-hexene insertion rate is lower than that without a substituent. In addition, the polymers showed high molecular weights (1.5–2.4 × 10^5^ g mol^−1^) and narrow molecular weight distributions (1.62–1.89). The obtained polymers were also verified to be amorphous copolymers and had high thermal stability, good solubility and optical transparency.

## Abstract 2

Ni(ii) complexes were successfully synthesized and characterized, and were highly active toward (co)polymerization of norbornene, and the 1-hexene content could be controlled.

## Graphical abstract

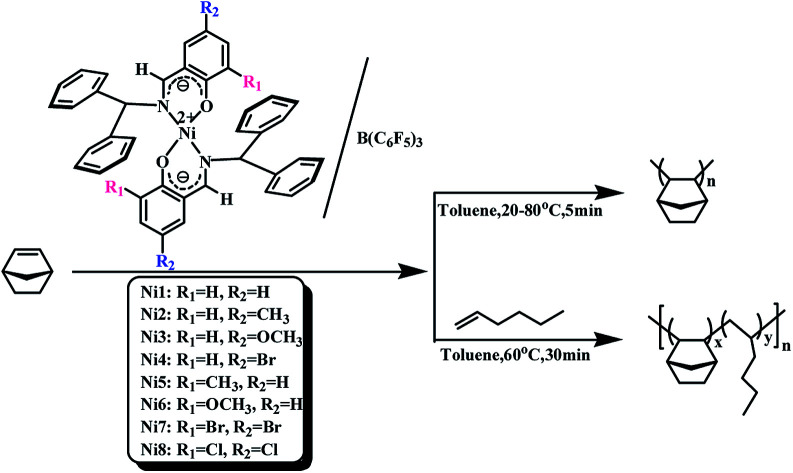

## 1. Introduction

Over the last few decades, polyolefin materials as a good substitute for engineering plastics and optical device apparatus attracted much attention in the scientific community and industry.^1–3^ Norbornene and its derivatives can be very easy to polymerize because of the presence of their bicyclic structures. There are three polymerization methods: ring-opening metathesis polymerization (ROMP),^4–8^ cationic or radical polymerization,^9^ and vinyl-addition polymerization.^10–17^ The polymers obtained by the vinyl addition polymerization method had high thermal stability, high optical permeability, a low dielectric constant, low moisture absorption and other excellent properties and were widely used in micro electronics and optics. However, the further development of norbornene is hampered by its poor bond properties, low solubility and poor mechanical properties. At present, the introduction of polar groups^18–20^ or flexible chains^21–25^ on the norbornene backbone is the most effective means to solve this problem. In order to achieve this goal, it is important to synthesize some efficient catalysts. It is well-known that conventional Ziegler–Natta catalysts^26,27^ and metallocene catalysts^28–30^ have many disadvantages due to their polar centers as early transition metals, for instance, poor tolerance to polar groups and large amounts of cocatalyst. However, owing to the lower oxophilicity, single active site and resistance toward deactivation by polar functionalities, the design of late-transition-metal catalysts has attracted widespread interest.^31–35^ The usual late-transition-metal catalysts are Ni(ii) and Pd(ii) complexes. Comprehensive investigations had approved Ni(ii) complexes because of their superior catalytic performance, polymer properties and their expounded reaction mechanisms.

Norbornene is a typical cycloolefin with many advantages of cycloolefins, but due to the presence of bicyclic skeletons in the polymer, it is brittle and has a small elongation at break. In order to improve its defects, the copolymer obtained by copolymerizing norbornene with α-olefin^22,23,36^ not only can reserve the heat resistance and dimensional stability of polynorbornene, but also has excellent optical properties, good processing and solubility, which has attracted scholars's attention at both home and abroad. For example, Kaminsky group^37^ achieved the copolymerization of norbornene with ethylene using a metallocene catalyst. The results showed that the glass transition temperature decreases with the increase of ethylene content in the copolymer, and the flexibility and processing performance of the polymer can be improved. Shiono group^38^ achieved the copolymerization of norbornene with long-chain alpha-olefins (1-hexene, 1-octene and 1-decene) through a titanium metal catalyst. And the catalyst afforded low molecular weight distribution, high light transmittance (up to 90%) and excellent processing performance.

In this study, a series of salicylaldimine ligands, (3-R_1_)(5-R_2_)C_6_H_2_(OH)CHNCH(C_6_H_5_)_2_ (R_1_ = H, R_2_ = H, L1; R_1_ = H, R_2_ = CH_3_, L2; R_1_ = H, R_2_ = OCH_3_, L3; R_1_ = H, R_2_ = Br, L4; R_1_ = CH_3_, R_2_ = H, L5; R_1_ = OCH_3_, R_2_ = H, L6; R_1_ = Br, R_2_ = Br, L7; R_1_ = Cl, R_2_ = Cl, L8) (L1–L8) and the corresponding bis-(salicylaldehyde-benzhydrylimino)nickel complexes (Ni1–Ni8) were synthesized, and the catalytic activities of complexes Ni1–Ni8 with only B(C_6_F_5_)_3_ (ref. 39–42) as cocatalyst in norbornene homopolymerization were investigated. In addition, the Ni1 and Ni2 complexes for norbornene and 1-hexene copolymerization were also investigated. Finally, the properties of polynorbornene were improved by introducing flexible chain, and the structure, thermal stability and optical properties of the copolymers were also characterized.

## 2. Experimental

All the operations involving air- and moisture-sensitive chemical compounds were performed under an atmosphere of purified and dried nitrogen using standard vacuum-line, Schlenk or glovebox techniques.

### 2.1 Materials

The required tetrahydrofuran (THF) and toluene were dried over sodium/benzophenone for 48 h and distilled under dry nitrogen atmosphere. Norbornene (NB) was purchased from Alfa Aesar and purified by drying over metallic sodium and distilling under dry nitrogen atmosphere, then used as a solution (0.4 g mL) in toluene. 1-Hexene was purchased from Aladdin and the inhibitor was removed by washing three times with aqueous sodium hydroxide solution (5.0 wt%), and then distillation over CaH_2_ under dry nitrogen atmosphere at a reduced pressure. Other chemical reagents available on the market are purchased and used without purification.

### 2.2 Characterization

The single crystal intensity data for the catalysts were collected with a Bruker Smart APEX II CCD system. The ^1^H NMR and ^13^C NMR spectra of the nickel complexes and copolymers were gained by a Bruker ARX 600 NMR spectrometer at ambient temperature with CDCl_3_ as solvent and tetramethylsilane (TMS) as an internal reference. The Fourier transform infrared (FT-IR) spectra were recorded by a Shimadzu IR Prestige-21 FTIR spectrophotometer. The molecular weight and molecular weight distribution of the polymers samples were gained by gel permeation chromatography (GPC, PL-GPC220), with a refractive index detector, chloroform as a solvent with a flow rate of 0.5 mL min^−1^ and detector at 40 °C. Thermal gravimetric analysis (TGA) was carried out with a TAQ600 SDT for thermogravimetry from room temperature to 650 °C at a rate of 20 °C min^−1^ under nitrogen atmosphere. The wide-angle X-ray diffraction (XRD) curves of the polymers were gained with a Bruker D8 Focus X-ray diffractometer, with monochromatic radiation at a wavelength of 1.54 Å at a scanning rate of 2° min^−1^. Scanning was carried out with 2*θ* ranging from 3° to 50°.

### 2.3 Crystal structure determination

The X-ray diffraction data of single crystals of the complexes were obtained with the *ω*−2*θ* scan mode of a Bruker Smart APEX II CCD diffractometer with graphite-monochromated Mo Kα radiation (*λ* = 0.71073 nm). The structures were worked out using direct methods, and further refinements with full-matrix least-squares on *F*^2^ were gained using the SHELXTL program package. All non-hydrogen atoms were refined anisotropically.

### 2.4 Syntheses of pro-ligands (L1–L8) and nickel complexes (Ni1–Ni8)

**Scheme 1:**
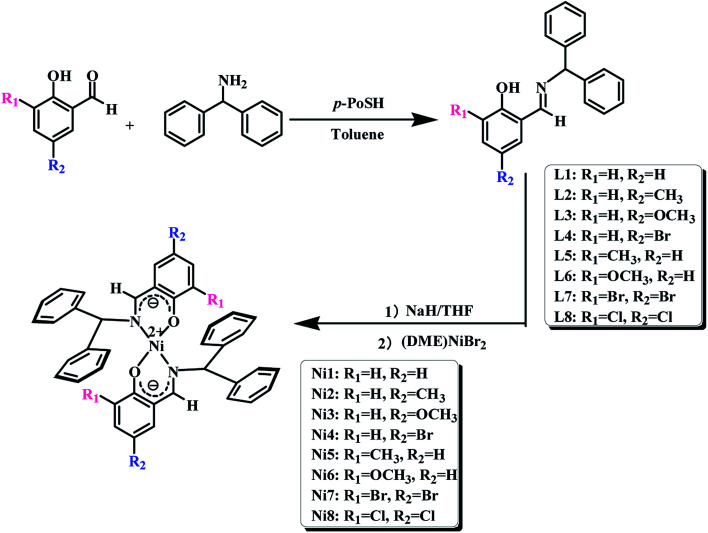
The synthetic routes of salicylaldehyde-benzhydrylimine ligands and bis-(salicylaldehyde-benzhydrylimino)nickel complexes.

The syntheses and structures of the pro-ligands and nickel complexes were outlined in Scheme 1.

The typical synthetic means of the C_6_H_4_(OH)CHNCH(C_6_H_5_)_2_ (L1) was described as follows: under a nitrogen atmosphere, salicylaldehyde 1.22 g (0.01 mol), aminodiphenylmethane 1.83 g (0.01 mol) and a catalytic amount of *p*-toluenesulfonic acid (*p*-TsOH) were successively added to a 250 mL reaction flask equipped with a magnetic stirrer, then added to 150 mL toluene. The mixture was refluxed for 24 h. The resulting water during the reaction was removed by using a Dean–Stark apparatus in the form of a water–toluene azeotrope. The crude product were further purified by silica column chromatography with *n*-hexane and ethyl acetate (20/1 in v/v) and were crystallized from a mixture of the *n*-hexane and ethyl acetate to get L1 as yellow crystals. Yield: 1.96 g (68.3%). ^1^H NMR (CDCl_3_, *δ*, ppm): 13.51 (s, 1H, OH); 8.47 (s, 1H, –C=N–H); 7.24–7.58 (m, 10H, 2C_6_H_5_); 6.86–7.24 (m, 3H, C_6_H_4_); 5.62 (s, 1H, C_13_H_11_). FT-IR (KBr): 3343.22(w), 2928.01(w), 2762.30(w), 1725.69(w), 1661.36 (*vs.*, *ν*_C=N_), 1592.06(s), 1516.49(w), 1433.45(w), 1300.56(w).

The other salicylaldimine ligands (L2–L8) were synthesized and characterized according to the method for L1 synthesizing, and yellow crystals were also obtained.

The typical synthetic procedure for Ni{(3-H)(5-H)C_6_H_2_(O)CHNCH(C_6_H_5_)_2_}_2_ (Ni1) was described as follow: ligand L1 (0.574 g, 0.002 mol) was added to a 100 mL Schlenk flask equipped with a magnetic stirrer, 0.06 mol of 60% sodium hydride was added to the reaction flask, and finally 20 mL of freshly distilled tetrahydrofuran solution was added. The mixed solution was stirred at room temperature for 8 hours for deprotonation. When the solution changed from yellow to orange, the solvent was removed *in vacuo*. Then, (DME)NiBr_2_ (1.0 mmol) and freshly distilled dichloromethane (20 mL) were added and stirred for 12 hours at room temperature. The dark-green crystal Ni1 complex was crystallized from dichloromethane in 70% yield. ^1^H NMR (CDCl_3_, *δ*, ppm): 8.45 (s, 2H, 2C–N–H); 0.97–1.49 (m, 20H, 4C_6_H_5_); 7.28–7.69 (m, 8H, 2C_6_H_4_); 5.62 (s, 2H, 2C_13_H_11_). ^13^C NMR (CDCl_3_, *δ*, ppm): 164.90, 132.61, 131.19, 129.48, 129.04, 127.43, 126.90, 118.85, 29.59. Analysis calculated for Ni1: C, 75.94; H, 5.75; N, 4.32; found: C, 76.01; H, 5.78; N, 4.33.

The other Ni{(3-R_1_)(5-R_2_)C_6_H_2_(O)CHNCH(C_6_H_5_)_2_}_2_ (Ni2–Ni8) were synthesized and characterized according to the method for synthesizing Ni1, and dark-green crystals were also obtained.

### 2.5 Polymerization of norbornene

**Scheme 2:**
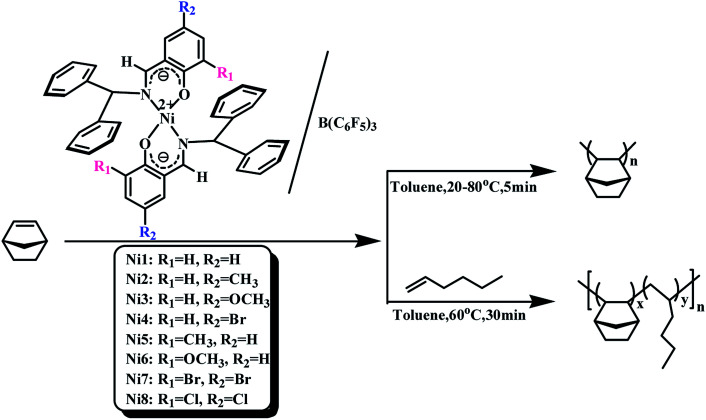
Norbornene homopolymerization catalysed by Ni1–Ni8/B(C_6_F_5_)_3_ and copolymerization of norbornene and 1-hexene catalyzed by Ni1 and Ni2/B(C_6_F_5_)_3_.

All manipulations were carried out under a dry nitrogen atmosphere. The polymerization process was as follows: the appropriate B(C_6_F_5_)_3_ standard solution and a certain amount of toluene were added to the two-necked round bottom flask, then the appropriate amount toluene solution of norbornene (NB) (0.4 g mL^−1^) and the catalyst standard solution (5 × 10^−3^ mol L^−1^) were injected into the stirred solution in order. The reaction mixture was continuously stirred at the set temperature. The reaction was terminated by the addition of a mixed solution of hydrochloric acid/methanol (v/v = 1/9). Finally, the resulting polymers were collected by filtration at room temperature and were dried in a vacuum at 50 °C until constant weight. The procedure was shown in Scheme 2.

### 2.6 Copolymerization of norbornene and 1-hexene

The copolymerization process was as follows: norbornene standard solution (0.4 g mol^−1^), 1-hexene and toluene were added to a 100 mL two-necked round bottom flask under nitrogen atmosphere, and then a standard solution of B(C_6_F_5_)_3_ (5 × 10^−2^ mol L^−1^) and complexes (5 × 10^−3^ mol L^−1^) were syringed into the well-stirred solution. The reaction mixture was continuously stirred for 1 h at 60 °C. The reaction was terminated by the addition of a mixed solution of hydrochloric acid/methanol (v/v = 1/9). The resulting polymers were collected by filtration at room temperature and were purified by trichloromethane/methanol reverse precipitation. Finally, the polymers were dried in a vacuum at 50 °C until constant weight. The procedure was shown in Scheme 2.

## 3. Results and discussion

### 3.1 Crystal structure

**Fig. 1:**
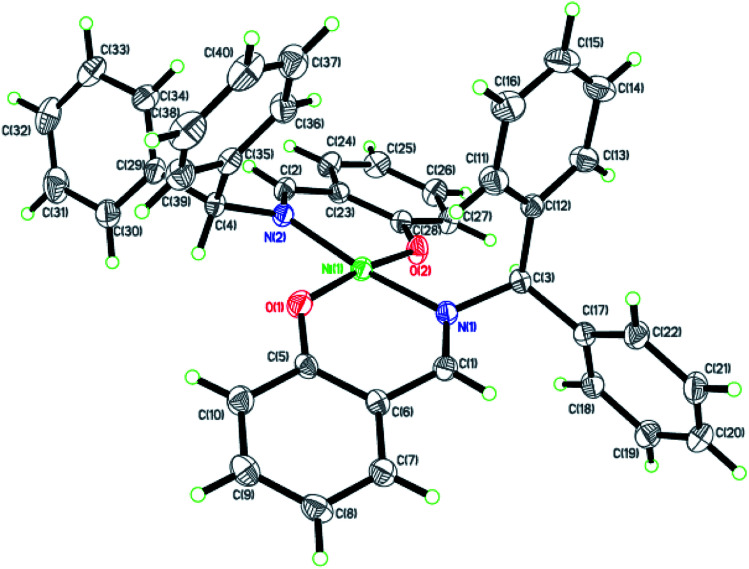
ORTEP plots of Ni1 with thermal ellipsoids at the 30% probability level showing the atom-labeling scheme. Hydrogen atoms and solvent have been omitted for clarity.

**Fig. 2:**
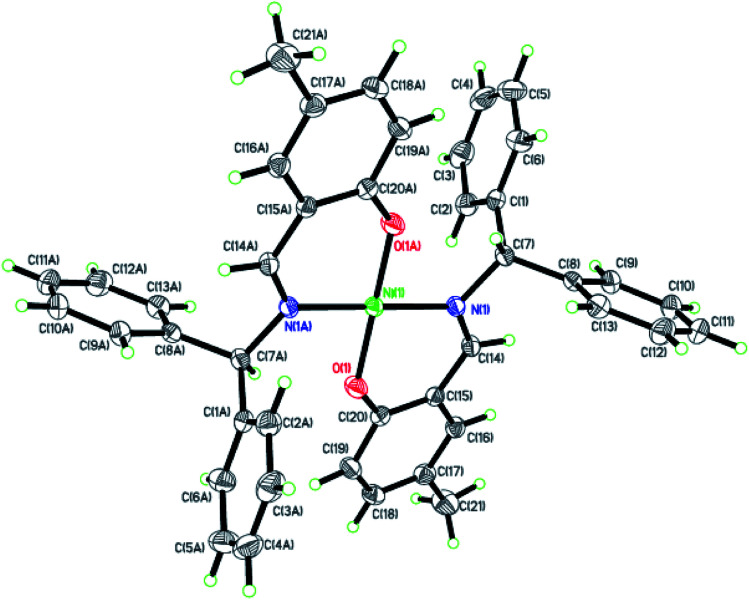
ORTEP plots of Ni2 with thermal ellipsoids at the 30% probability level showing the atom-labeling scheme. Hydrogen atoms and solvent have been omitted for clarity.

**Fig. 3:**
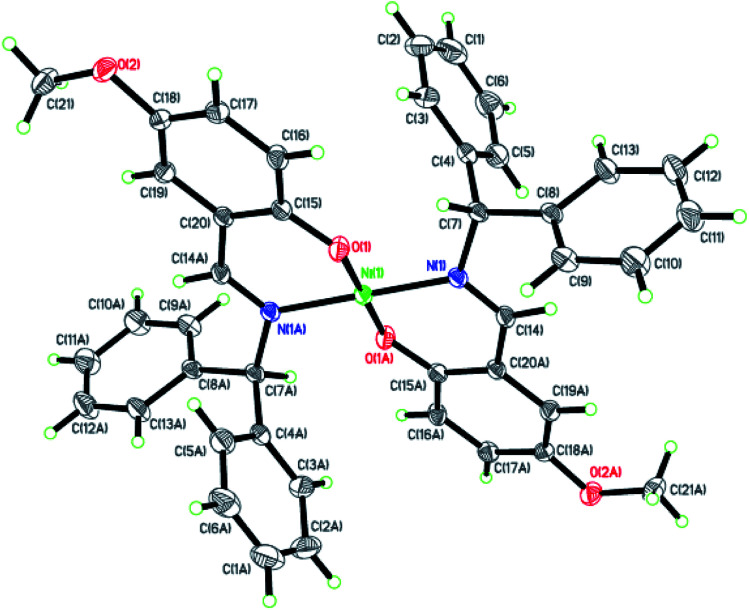
ORTEP plots of Ni3 with thermal ellipsoids at the 30% probability level showing the atom-labeling scheme. Hydrogen atoms and solvent have been omitted for clarity.

**Fig. 4:**
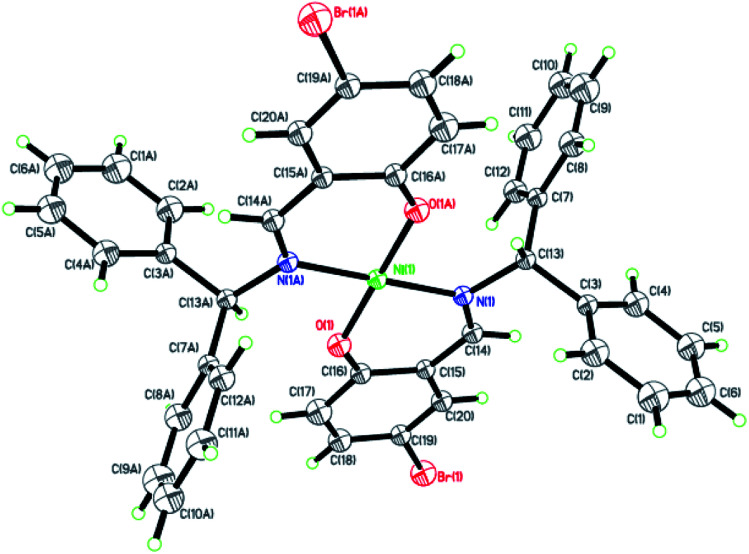
ORTEP plots of Ni4 with thermal ellipsoids at the 30% probability level showing the atom-labeling scheme. Hydrogen atoms and solvent have been omitted for clarity.

**Fig. 5:**
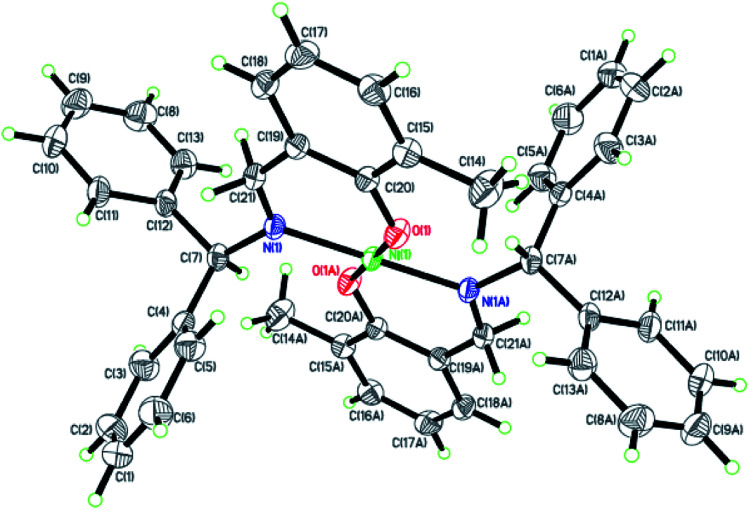
ORTEP plots of Ni5 with thermal ellipsoids at the 30% probability level showing the atom-labeling scheme. Hydrogen atoms and solvent have been omitted for clarity.

**Fig. 6:**
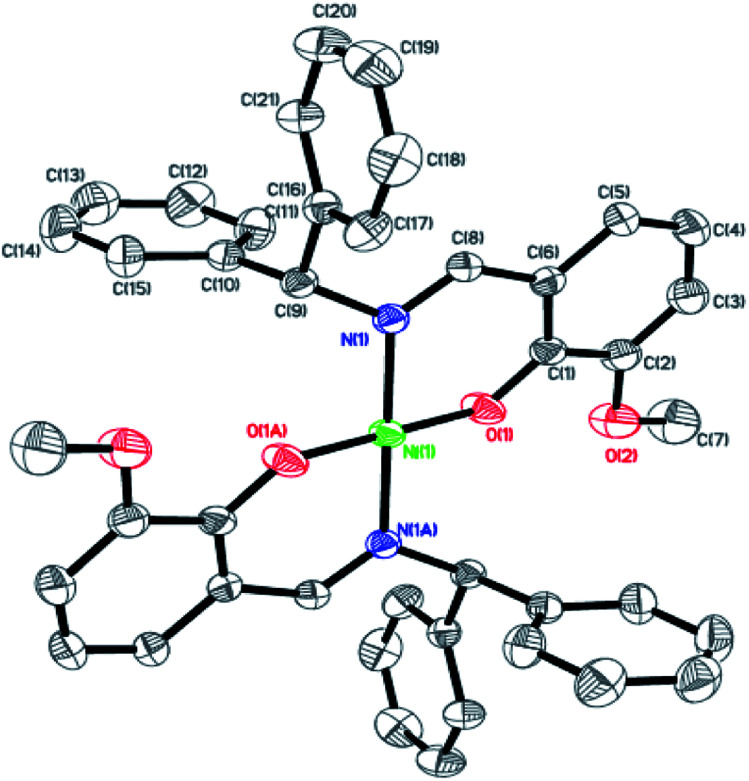
ORTEP plots of Ni6 with thermal ellipsoids at the 30% probability level showing the atom-labeling scheme. Hydrogen atoms and solvent have been omitted for clarity.

**Fig. 7:**
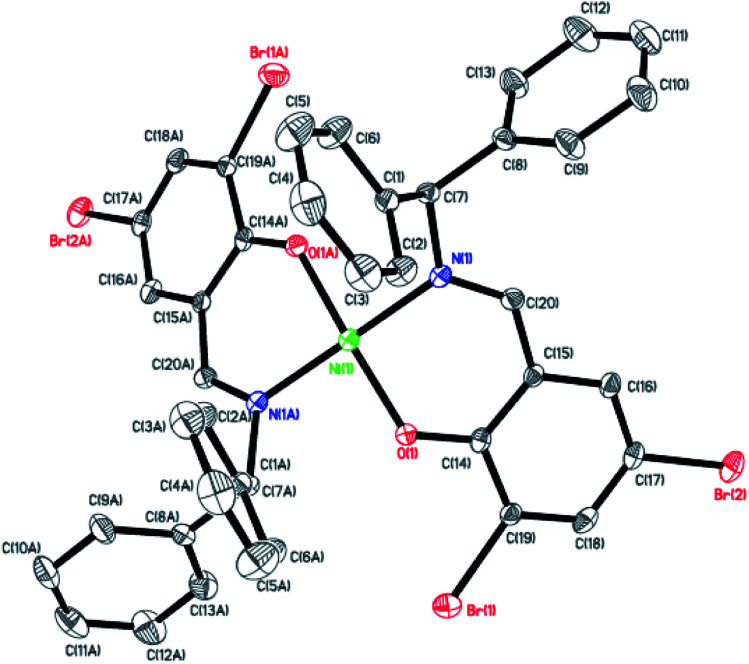
ORTEP plots of Ni7 with thermal ellipsoids at the 30% probability level showing the atom-labeling scheme. Hydrogen atoms and solvent have been omitted for clarity.

**Fig. 8:**
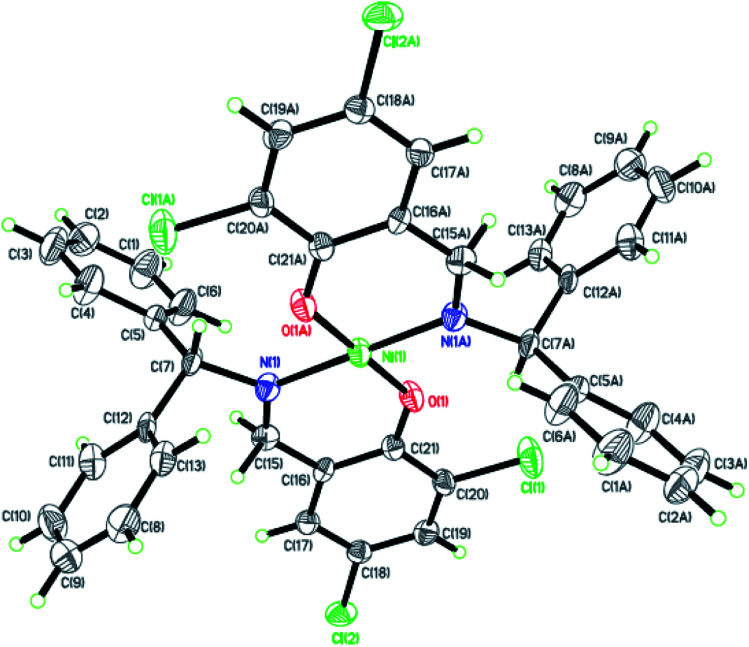
ORTEP plots of Ni8 with thermal ellipsoids at the 30% probability level showing the atom-labeling scheme. Hydrogen atoms and solvent have been omitted for clarity.

Dark-green single crystals of complexes suitable for single crystal X-ray diffraction were gained by slow diffusion toluene into dichloromethane solutions. The crystallographic data were summarized in Tables 1 and 2 and Table S1 and S2.† The ORTEP plots of Ni1–Ni8 were shown in Fig. 1–8, respectively. CIF data of Ni1– Ni8 are available as ESI.† In the solid state, all the complexes are both mononuclear and four coordinate and adopt geometries best described as square planar about each nickel center, having slight distortions from idealized geometry and are quiet similar to some known nickel(ii) complexes.^19^ In fact, the Ni–O bond lengths of Ni1 (1.816(4) Å), Ni2 (1.8295(12) Å), Ni3 (1.831(2) Å), Ni4 (1.8480(14) Å), Ni5 (1.826(8) Å), Ni6 (1.8298(16) Å), Ni7 (1.840(2) Å) and Ni8 (1.846(3) Å) are shorter than the Ni–N bond lengths of Ni1 (1.927(4) Å), Ni2 (1.9300(13) Å), Ni3 (1.916(2) Å), Ni4 (1.9261(16) Å), Ni5 (1.921(7) Å), Ni6 (1.919(2) Å), Ni7 (1.899(3) Å) and Ni8 (1.931(3) Å). The pro-ligands are very stable structure and the catalysts structure are also stable and symmetrical, therefore there are very advantageous for stabilizing the activity of the catalyst. In addition, the ligand electronic effect generate different charge distribution on the nickel metal atom, and the catalytic activity markedly increased with an increase in the electrophilicity of the nickel metal center.

**Table 1:** Crystal data and structure refinement details for Ni1–Ni4

	Ni1	Ni2	Ni3	Ni4
Empirical formula	C_40_H_32_N_2_NiO_2_	C_42_H_36_N_2_NiO_2_	C_42_H_36_N_2_NiO_4_	C_40_H_30_Br_2_N_2_NiO_2_
Formula weight	631.39	659.44	772.31	789.15
Crystal color	Dark-green	Dark-green	Dark-green	Dark-green
Temperature (K)	296(2)	296(2)	296(2)	296(2) K
Wavelength (Å)	0.71073	0.71073	0.71073	0.71073 A
Crystal system	Monoclinic	Monoclinic	Monoclinic	Monoclinic
Space group	*P*2(1)/*n*	*P*2(1)/*n*	*P*2(1)/*n*	*P*2(1)/*n*
*a* (Å)	17.482(8)	11.0313(11)	9.960(7)	17.8459(14)
*b* (Å)	10.366(5)	11.2443(11)	24.935(16)	9.8816(7)
*c* (Å)	18.537(8)	13.3797(13)	8.698(6)	20.2608(16)
*α* (deg)	90	90	90	90
*β* (deg)	110.601(5)	99.094(10)	104.781(8)	110.6450(10)
*γ* (deg)	90	90	90	90
Volume (Å^3^)	3144(2)	1638.7(3)	2089(2)	3343.5(4)
*Z*	4	2	2	4
*D*_cal_ a (g m^−3^)	1.334	1.336	1.363	1.568
Abs coeff (mm^−1^)	0.656	0.632	0.764	3.009
*F*(000)	1320	692	884	1592.0
Crystal size (mm)	0.80 × 0.30 × 0.30	0.25 × 0.22 × 0.15	0.25 × 0.23 × 0.20	0.25 × 0.20 × 0.15
*θ* range (deg)	2.35 to 26.63	2.23 to 25.50	2.27 to 27.61	2.15 to 27.76
Limiting indices	−20 ≤ *h* ≤ 21, −12 ≤ *k* ≤ 12, −23 ≤ *l* ≤ 23	−13 ≤ *h* ≤ 13, −13 ≤ *k* ≤ 13, −14 ≤ *l* ≤ 16	−12 ≤ *h* ≤ 12, −32 ≤ *k* ≤ 32, −11 ≤ *l* ≤ 11	−23 ≤ *h* ≤ 22, −12 ≤ *k* ≤ 12, −26 ≤ *l* ≤ 26
Max. and min. transmission	0.821 and 0.790	0.9111 and 0.8580	0.858 and 0.826	0.637 and 0.491
Refinement method	Full-matrix least-squares on *F*^2^	Full-matrix least-squares on *F*^2^	Full-matrix least-squares on *F*^2^	Full-matrix least-squares on *F*^2^
Data/restraints/parameters	6425/0/412	3050/0/215	4796/30/270	7821/0/187
Goodness-of-fit on *S* (*F*^2^)^a^	1.098	1.070	1.035	2.001
Final *R* indices [*I* > 2*σ*(*I*)]	*R*1 = 0.0601, w*R*2 = 0.1583	*R*1 = 0.0286, w*R*2 = 0.0836	*R*1 = 0.0541, w*R*2 = 0.1312	*R*1 = 0.1327, w*R*2 = 0.3291
*R* indices (all data)	*R*1 = 0.0912, w*R*2 = 0.1784	*R*1 = 0.0317, w*R*2 = 0.0864	*R*1 = 0.1042, w*R*2 = 0.1469	*R*1 = 0.2086, w*R*2 = 0.3587
Largest diff peak and hole (e Å^−3^)	0.509 and −1.087	0.263 and −0.269	0.44 and −0.40	4.723 and −5.169

a
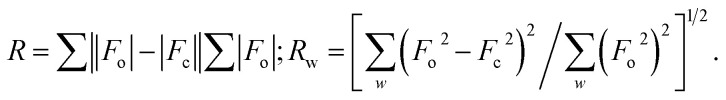

**Table 2:** Crystal data and structure refinement details for Ni5–Ni8

	Ni5	Ni6	Ni7	Ni8
Empirical formula	C_42_H_38_N_2_NiO_2_	C_42_H_36_N_2_NiO_4_	C_40_H_28_Br_4_N_2_NiO_2_	C_40_H_28_Cl_4_N_2_NiO_2_
Formula weight	661.43	691.44	946.93	769.13
Crystal color	Dark-green	Dark-green	Dark-green	Dark-green
Temperature (K)	296(2) K	296(2)	293(2)	296(2)
Wavelength (Å)	0.71073 A	0.71073	0.71073	0.71073
Crystal system	Monoclinic	Monoclinic	Monoclinic	Triclinic
Space group	*P*2(1)/*n*	*P*2(1)/*n*	*C*2/*c*	*P*1̄
*a* (Å)	7.6601(6)	10.243(4)	24.331(8)	9.1018(10)
*b* (Å)	13.1771(10)	16.148(6)	8.402(3)	13.8097(15)
*c* (Å)	16.4976(13)	*c* = 12.283(5)	20.590(6) A	16.5217
*α* (deg)	90	90	90	73.923(2)
*β* (deg)	98.9180(10)	93.351(4)	121.627(3)	74.3970(10)
*γ* (deg)	90	90	90	81.837(2)
Volume (Å^3^)	1645.1(2)	2028.2(14)	3584(2)	1916.8(4)
*Z*	2	2	4	1
*D*_cal_ a (g m^−3^)	1.335	1.132	1.755	1.461
Abs coeff (mm^−1^)	0.630	0.517	5.039	0.829
*F*(000)	696	724	1864	872.0
Crystal size (mm)	0.25 × 0.20 × 0.15	0.25 × 0.22 × 0.20	0.25 × 0.23 × 0.20	0.25 × 0.20 × 0.15
*θ* range (deg)	2.50 to 27.59	2.36 to 25.50	2.32 to 27.66	2.27 to 27.42
Limiting indices	−9 ≤ *h* ≤ 9, −15 ≤ *k* ≤ 17, −21 ≤ *l* ≤ 21	−12 ≤ *h* ≤ 12, −19 ≤ *k* ≤ 19, −14 ≤ *l* ≤ 14	−28 ≤ *h* ≤ 31, −10 ≤ *k* ≤ 10, −26 ≤ *l* ≤ 26	−11 ≤ *h* ≤ 11, −17 ≤ *k* ≤ 17, −20 ≤ *l* ≤ 21
Max. and min. transmission	0.910 and 0.860	0.9036 and 0.8816	0.365 and 0.295	0.883 and 0.820
Refinement method	Full-matrix least-squares on *F*^2^	Full-matrix least-squares on *F*^2^	Full-matrix least-squares on *F*^2^	Full-matrix least-squares on *F*^2^
Data/restraints/parameters	3798/0/214	3776/0/224	4128/0/222	8567/0/490
Goodness-of-fit on *S* (*F*^2^)^a^	1.142	1.135	1.264	1.167
Final *R* indices [*I* > 2*σ*(*I*)]	*R*1 = 0.0423, w*R*2 = 0.1371	*R*1 = 0.0400, w*R*2 = 0.1257	*R*1 = 0.0434, w*R*2 = 0.0562	*R*1 = 0.0702, w*R*2 = 0.0762
*R* indices (all data)	*R*1 = 0.0544, w*R*2 = 0.1499	*R*1 = 0.0462, w*R*2 = 0.1353	*R*1 = 0.1206, w*R*2 = 0.0651	*R*1 = 0.1887, w*R*2 = 0.0905
Largest diff peak and hole (e Å^−3^)	0.581 and −0.724	0.309 and −0.197	0.52 and −0.53	0.711 and −0.541

a
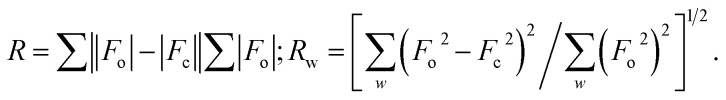

### 3.2 Polymerization of norbornene

A series of nickel complexes were tested with B(C_6_F_5_)_3_ (ref. 43 and 44) as cocatalyst in toluene and the detailed data were summarized in Table 3. In order to determine the most suitable norbornene homopolymerization conditions, the catalytic performance of Ni1 was investigated by changing the NB/Ni molar ratios, the B/Ni molar ratios, and the polymerization temperature. From Table 3 we found that the polymer yields and catalytic activities depended on the reaction conditions, and the NB/Ni molar ratio at 2000/1, B/Ni molar ratio at 10/1 and the polymerization at 60 °C were the best polymerization conditions. All catalysts exhibited very high activity (up to 10^6^ g_polymer_ mol_Ni_^−1^ h^−1^), but there are also certain differences between the different catalysts. It can be seen that when the R2 substituent at the 5-position of the salicylaldimino aromatic ring is a strong electron-withdrawing group (–Br), the catalytic activity is higher than that at the corresponding position for the electron-donating group (–CH_3_ or –OCH_3_) (Ni4 > Ni1, Ni2, Ni3), indicating that the electron-withdrawing group on the salicylaldimino aromatic ring enhances the activity of the catalyst. If the salicylaldimino aromatic ring contains the same electron-donating group (–CH_3_ or –OCH_3_), the 5-position substituent (R2) has a higher activity than the 3-position substituent (R1) (Ni2 > Ni5, Ni3 > Ni6), which most important reason may be that the 3-position substituent (R1) of the salicylaldimino aromatic ring increased the steric hindrance effect of the catalyst, and this had little effect on the chain transfer reaction of polynorbornene. If the 3-position (R1) and 5-position (R2) both contain strong electron-withdrawing groups (–Br or –Cl), the catalytic activity is higher than those of the 3-position (R1) or the 5-position (R2) (Ni7 > Ni4), which indicated that the activity of the catalyst may be the result of the synergistic effect of the electronic and steric effects of the substituent.^39,45^ These results indicate that the strong electron-withdrawing group facilitates the increase of the activity of the catalyst, and the reactivity and the yield of the polymer increase with the electron-withdrawing ability of the salicylaldimino aromatic ring substituent.

**Table 3:** Homopolymerization of norbornene catalyzed by Ni1–Ni8 combined with B(C_6_F_5_)_3_^a^

Ni complexes	NB/Ni (mol mol^−1^)	B/Ni (mol mol^−1^)	*T*_p_, °C	Reaction time (min)	PNB Yield (%)	Activity^b^
Ni1	1000	10	60	5	60.42	0.68
Ni1	2000	10	60	5	70.25	1.62
Ni1	3000	10	60	5	42.61	1.44
Ni1	4000	10	60	5	27.67	1.25
Ni1	2000	5	60	5	29.63	0.67
Ni1	2000	20	60	5	58.35	1.32
Ni1	2000	10	20	10	40.66	0.46
Ni1	2000	10	40	10	62.52	0.71
Ni1	2000	10	80	5	66.37	1.50
Ni2	2000	5	60	5	34.67	0.78
Ni2	2000	10	60	5	73.35	1.65
Ni2	2000	20	60	5	61.85	1.39
Ni3	2000	5	60	5	32.34	0.73
Ni3	2000	10	60	5	71.25	1.61
Ni3	2000	20	60	5	60.73	1.37
Ni4	2000	5	60	5	37.31	0.84
Ni4	2000	10	60	5	78.45	1.77
Ni4	2000	20	60	5	67.72	1.53
Ni5	2000	5	60	5	33.42	0.75
Ni5	2000	10	60	5	72.21	1.63
Ni5	2000	20	60	5	61.25	1.38
Ni6	2000	5	60	5	30.48	0.68
Ni6	2000	10	60	5	69.86	1.58
Ni6	2000	20	60	5	58.67	1.32
Ni7	2000	5	60	5	38.42	0.87
Ni7	2000	10	60	5	85.67	1.93
Ni7	2000	20	60	5	70.26	1.59
Ni8	2000	5	60	5	40.45	0.91
Ni8	2000	10	60	5	91.78	2.07
Ni8	2000	20	60	5	73.65	1.66

a Reaction conditions: *n*[Ni] = 5.0 × 10^−6^ mol; total volume toluene 10 mL.

b In units of 10^6^ g_polymer_ mol_Ni_^−1^ h^−1^.

### 3.3 Copolymerization of norbornene and 1-hexene

To further investigate the effect of the catalytic activity, Ni1 and Ni2 were applied to the copolymerization of norbornene (NB) and 1-hexene. The detailed data were summarized in Table 4 and were collected under similar conditions. It is apparent that the activities of Ni1 and Ni2 are significantly lower than those observed with norbornene homopolymerization. This may be related to the stronger binding of 1-hexene to the Ni center and the longer chain structure of 1-hexene relative to norbornene, delaying the rate of chain growth compared to norbornene homopolymerization. The incorporation levels of 1-hexene are also different, Ni1 displayed higher incorporation ability for 1-hexene and provided copolymers having higher 1-hexene contents (8.6–12.50 mol%) than Ni2 (7.9–11.40 mol%) under the same conditions. In addition, we learned that the substituents on salicylaldimino aromatic ring have a profound effect on the copolymerization reactivity of the catalyst system.^46^ It is well known that the methyl (–CH_3_) belongs to the electron donating group and can cause an increase in the electron cloud density on the salicylaldimino aromatic ring, but the results in Table 4 showed that the Ni2/B(C_6_F_5_)_3_ had higher activities (0.79–2.40 × 10^5^ g_polymer_ mol^−1^ h^−1^) than Ni1 (0.55–2.03 × 10^5^ g_polymer_ mol_Ni_^−1^ h^−1^) did. The main reason may be that the 5-position substituent (–CH_3_) of the salicylaldimino aromatic ring has a certain steric hindrance effect to shield the active central axis surface, on the one hand, it can reduce chain transfer and prevent chain termination, on the other hand, it can also inhibit the occurrence of ligand rearrangement and disproportionation reaction.^47,50^ Besides, with the 1-hexene flexible chain increases, the polymerization activity and the polymer yield decrease. The obtained polymers had good solubility and were soluble in common organic solvents (such as CHCl_3_, CH_2_Cl_2_, cyclohexane, THF) as well as chlorobenzene at ambient temperature, which exhibited better solubility than PNB did.

**Table 4:** Copolymerization of norbornene and 1-hexene catalyzed by Ni1–Ni2 combined with B(C_6_F_5_)_3_^a^

Ni complexes	NB/1-hexene (*n*/*n*)	Yield (%)	Activity (g_polymer_ mol_Ni_^−1^ h^−1^)	*M*_w_ b	*M*_w_/*M*_n_^b^	1-Hexene incorp^c^ (mol%)
Ni1	100/0	70.25	1.62 × 10^6^	Insoluble	—	0
Ni1	90/10	54.40	2.03 × 10^5^	2.13	1.66	8.36
Ni1	70/30	36.21	1.32 × 10^5^	1.99	1.68	10.10
Ni1	50/50	15.52	0.55 × 10^5^	1.45	1.66	12.50
Ni2	100/0	73.35	1.65 × 10^6^	Insoluble	—	0
Ni2	90/10	64.50	2.40 × 10^5^	2.39	1.89	7.98
Ni2	70/30	34.61	1.25 × 10^5^	2.04	1.64	9.45
Ni2	50/50	22.30	0.79 × 10^5^	1.48	1.62	11.40

a Reaction conditions: *n*[Ni] = 5.0 × 10^−6^ mol; *n*[NB] + *n*[1-hexene] = 0.01 mol; solvent: toluene; reaction time: 30 min; temperature: 60 °C; *n*[B]/*n*[Ni] = 10/1.

b Determined by GPC *vs.* polystyrene standards in CHCl_3_. In unites of 10^5^ g mol^−1^.

c Determined by ^1^HNMR spectroscopy in CDCl_3_.

### 3.4 GPC curves of copolymers

**Fig. 9:**
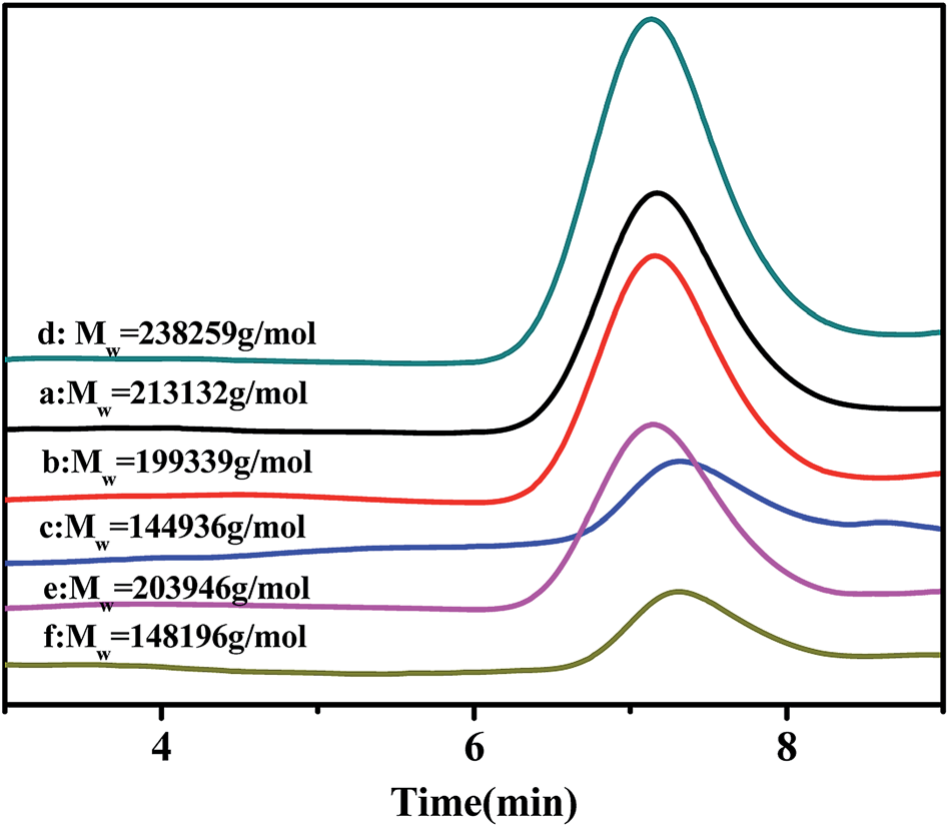
The GPC curves of poly(NB-*co*-1-hexene)s with (a) 8.36, (b) 10.10, (c) 12.50, (d) 7.98, (e) 9.45 (f) 11.40 mol% 1-hexene. (a), (b) and (c) obtained byNi1/B(C_6_F_5_)_3_ system, (d), (e) and (f) obtained by Ni2/B(C_6_F_5_)_3_ system respectively.

Gel permeation chromatography (GPC) curves of the poly(NB-*co*-1-hexene)s with different 1-hexene flexible chain incorporation ratios achieved by the Ni1 and Ni2/B(C_6_F_5_)_3_ systems are shown in Fig. 9. GPC measurements indicated that all the polymers had high molecular weight (*M*_w_) ranging from 1.45 × 10^5^ to 2.39 × 10^5^ g mol^−1^ and decreased with the increase of 1-hexene insertion rate. The distribution of molecular weight (PDI, *M*_w_/*M*_n_) is very narrow in the range of 1.62–1.89. In addition, the GPC curves for all polymers are all unimodal, indicating that the polymerization was initiated by a single active site, which established that the resulting polymer is copolymers rather than blends of homopolymers.

### 3.5 ^1^H and ^13^C NMR spectra of copolymers

**Fig. 10:**
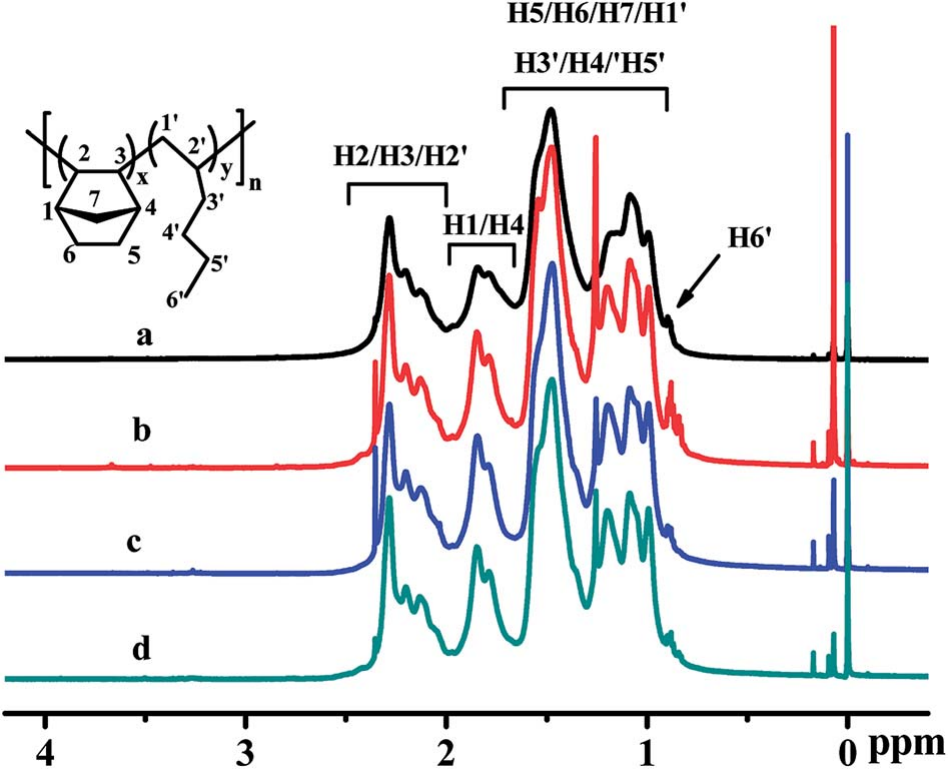
^1^H NMR spectra of poly(NB-*co*-1-hexene)s with (a) 10.10, (b) 12.50, (c) 9.45, (d) 11.40 mol% 1-hexene. (a) and (b) obtained by Ni1/B(C_6_F_5_)_3_ system, (c) and (d) obtained by Ni2/B(C_6_F_5_)_3_ system respectively.

The poly(NB-*co*-1-hexene)s were characterized by ^1^H NMR spectroscopy and the results were shown in Fig. 10, which demonstrated that the polymers were vinyl-addition type by the absence of the resonance of the proton hydrogen connected to the double bond at 5.3–6.0 ppm.^48^ From the ^1^HNMR spectra of Fig. 10, the characteristic peaks at 0.8–0.9 ppm could be assigned to the hydrogen corresponding to H6′, the characteristic peaks at 0.9–1.6 ppm could be attributed to the hydrogen corresponding to H5/H6/H7/H1′/H3′/H4′/H5′, the characteristic peaks at 1.6–2.0 ppm could be attributed to the hydrogen corresponding to H1/H4 and those at 2.0–2.3 ppm could be attributed to the hydrogen corresponding to H2/H3/H2′. The molar fractions of 1-hexene could be calculated^23^ by ^1^H NMR analyses and were 24.9, 22.4 and 11.3 mol%.

**Fig. 11:**
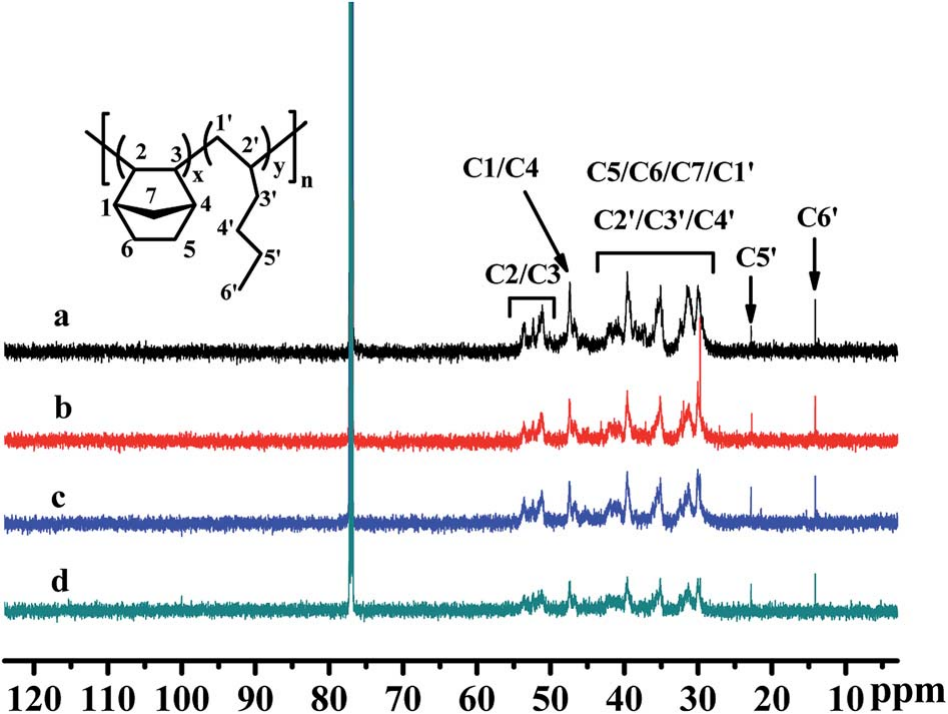
^13^C NMR spectra of poly(NB-*co*-1-hexene)s with (a) 10.10, (b) 12.50, (c) 9.45, (d) 11.40 mol% 1-hexene. (a) and (b) obtained by Ni1/B(C_6_F_5_)_3_ system, (c) and (d) obtained by Ni2/B(C_6_F_5_)_3_ system respectively.

The ^13^C NMR spectra of the poly(NB-*co*-1-hexene)s were shown in Fig. 11. The absorption peaks at 14.0 ppm and 22.9 ppm were attributed to the methyl C6′ and methylene C5′ on the alkyl carbon chain. The absorption peaks between 45.8–48.1 ppm and 50.3–54.3 ppm were attributed to methine C1/C4 and C2/C3 on norbornene, respectively. And the absorption peak of the copolymer between 28.8–42.8 ppm could be attributed to C5/C6/C7/C1′/C2′/C3′/C4′. In addition, the characteristic peak of ring-opening metathesis polymerization of norbornene was not found at 120 ppm, which further indicated that the copolymerization of norbornene and 1-hexene catalyzed by the Ni1 and Ni2/B(C_6_F_5_)_3_ system was performed *via* vinyl addition mechanism.

### 3.6 FTIR spectra of copolymers

**Fig. 12:**
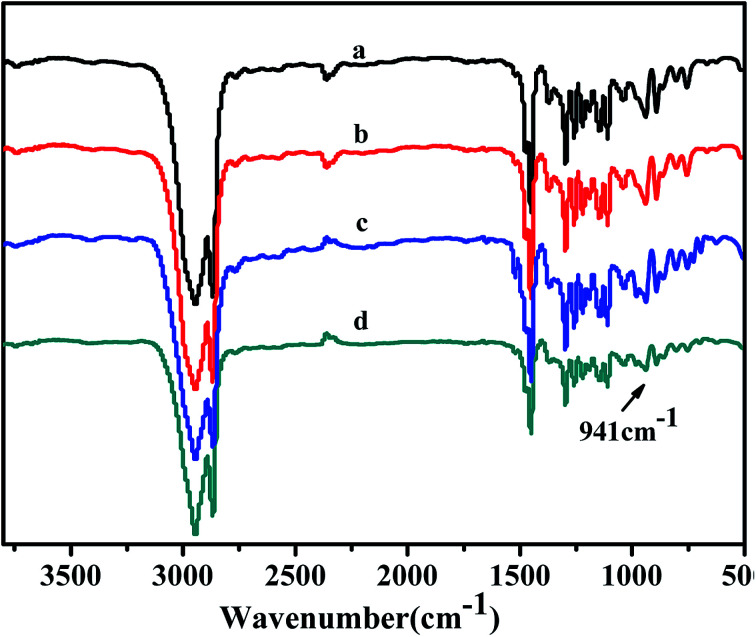
FT-IR spectra of poly(NB-*co*-1-hexene)s with (a) 10.10, (b) 12.50, (c) 9.45, (d) 11.40 mol% 1-hexene. (a) and (b) obtained by Ni1/B(C_6_F_5_)_3_ system, (c) and (d) obtained by Ni2/B(C_6_F_5_)_3_ system, respectively.

The structures of obtained copolymers were characterized by FTIR spectroscopy and shown in Fig. 12. All of the copolymers obtained from the FTIR spectrum show an absorption peak at 941 cm^−1^ could be assigned to the ring of bicyclo[2.2.1] heptane, as Kennedy and Makowski reported.^49^ There were no characteristic absorption peak at about 1620–1680 cm^−1^ and 960 cm^−1^ of the carbon–carbon double bond (C=C) of the structure of ROMP type PNB. In addition, there were also no absorptions peak at about 3050 cm^−1^ and 2100 cm^−1^ to the 1-hexene characteristic trans form of C=C and C–H bonds stretching. All these results shown that norbornene and 1-hexene were successfully copolymerized and further indicated 1-hexene had been inserted into the copolymers chains rather than simple blends.

### 3.7 TGA analyses of copolymers

**Fig. 13:**
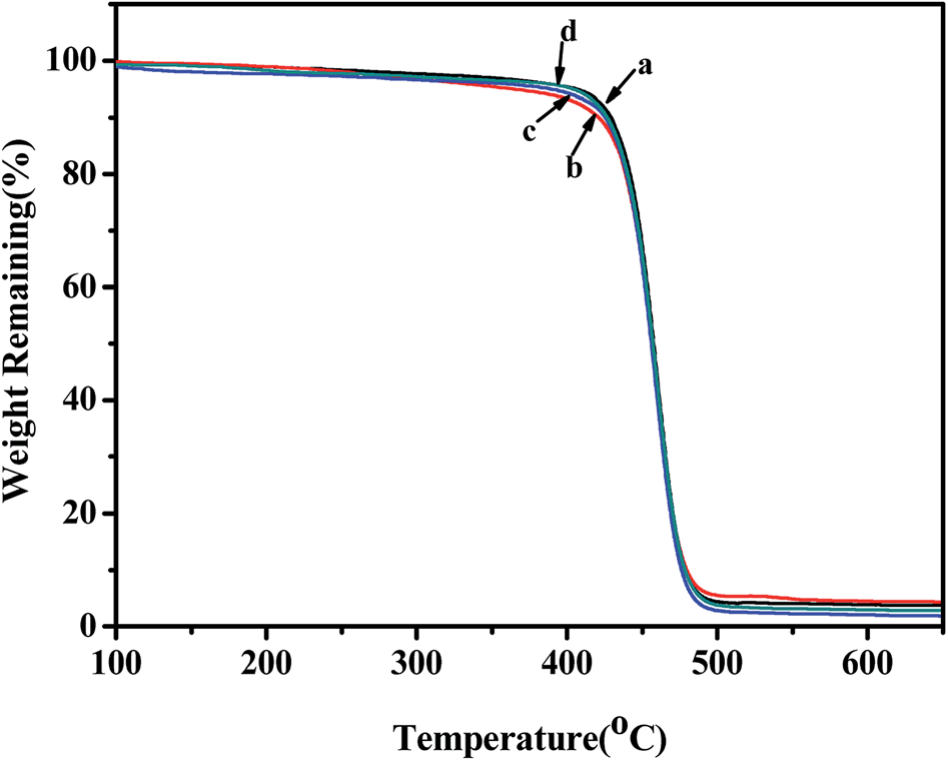
TGA curves of poly(NB-*co*-1-hexene)s with (a) 10.10, (b) 12.50, (c) 9.45, (d) 11.40 mol% 1-hexene. (a) and (b) obtained by Ni1/B(C_6_F_5_)_3_ system, (c) and (d) obtained by Ni2/B(C_6_F_5_)_3_ system, respectively.

The TGA curves of poly(NB-*co*-1-hexene) with different 1-hexene insertion rates gained with the Ni1 and Ni2/B(C_6_F_5_)_3_ catalytic system were shown in Fig. 13. The thermal properties of all the copolymers obtained from the TGA curve were similar and all began to decompose within the range of 350–450 °C. In addition, as the insertion of the 1-hexene content in the polymer chain increased, the thermal decomposition temperature decreased, but it still had good thermal stability.

### 3.8 WXRD analyses of the copolymers

**Fig. 14:**
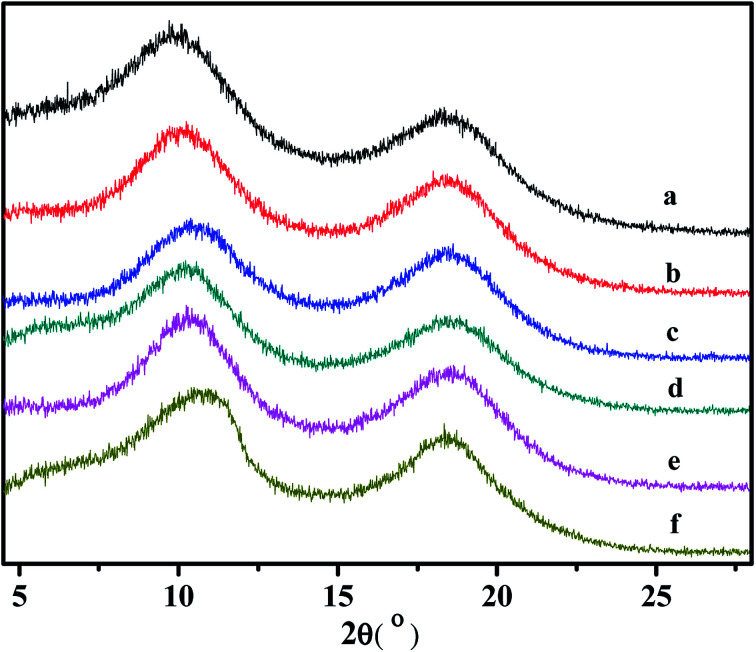
WXRD spectra of poly(NB-*co*-1-hexene)s with (a) 8.36%, (b) 10.10, (c) 12.50, (d) 7.98, (e) 9.45, (f) 11.40 mol% 1-hexene. (a), (b) and (c) obtained by Ni1/B(C_6_F_5_)_3_ system, (d), (e) and (f) obtained by Ni2/B(C_6_F_5_)_3_ system, respectively.

Some supporting information about the conformation of the poly(NB-*co*-1-hexene)s were gained with wide-angle X-ray diffraction (WAXD), as shown in Fig. 14. No Bragg diffraction appears in the spectral region of the crystal, so the polymers were noncrystalline. Two broad halos at 2 values of 10.42–10.58° and 18.04–18.28° were characteristic peaks for polynorbornene. When the catalytic system is changed, the insertion rate of 1-hexene is different, but the change of the diffraction peak is less obvious and the chain spacing of all the polymers is not much different. These all results shown that the packing density of the copolymers changed little when the catalytic system changed. The interchain distances were computed according to eqn (1) and amounted to be 8.50 and 4.77 Å, separately.1
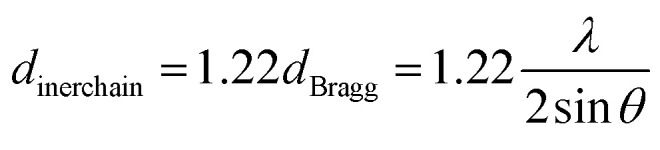


### 3.9 The transparency properties of the copolymer films

**Fig. 15:**
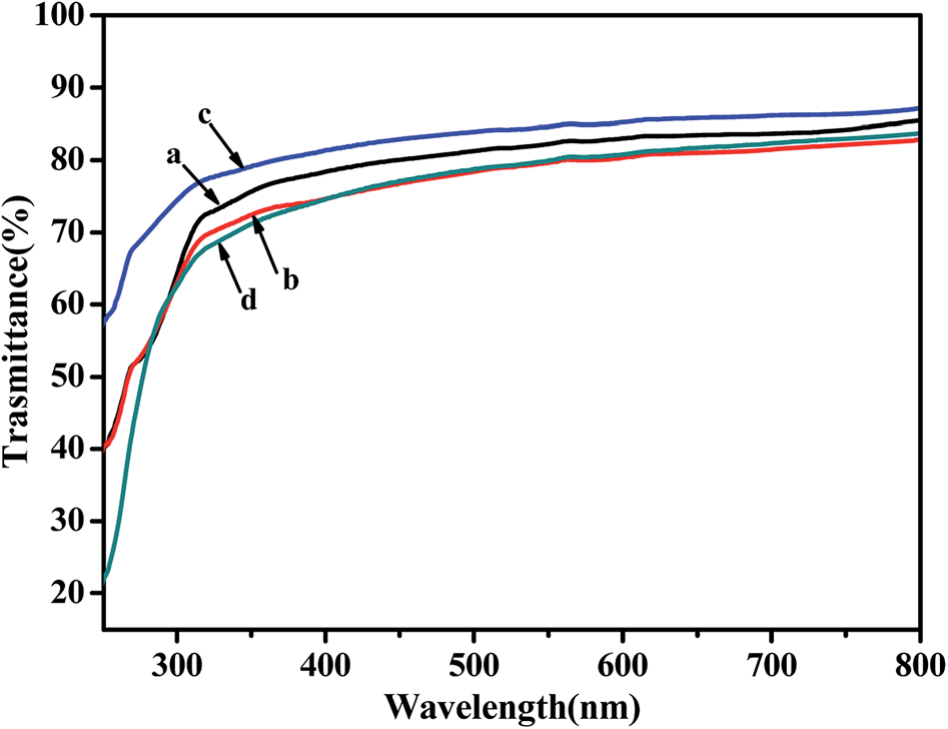
The UV-Vis curves of poly(NB-*co*-1-hexene)s membranes with (a) 10.10, (b) 12.50, (c) 9.45, (d) 11.40 mol% 1-hexene. (a) and (b) obtained by Ni1/B(C_6_F_5_)_3_ system, (c) and (d) obtained by Ni2/B(C_6_F_5_)_3_ system, respectively.

The UV-Vis curves of poly(NB-*co*-1-hexene)s films with different 1-hexene insertion rate were shown in Fig. 15. The spectrum shown that the transmittance of the copolymer film in the visible light range of 400–800 nm were all over 80%. Although the transmittance of the copolymers were lower than the polynorbornene (92%), the copolymer still had good light transmittance. In addition, as the insertion rate of 1-hexene in the copolymer was increased, the light transmittance of the polymer films were decreased, which indicated that the addition of 1-hexene had a negative effect on the light transparency of the copolymer films.

## 4. Conclusions

Eight bis-(salicylaldehyde-benzhydrylimino)nickel complexes with different electron groups (Ni1–Ni8) were synthesized and characterized by single crystal X-ray diffraction, and these catalysts achieved homopolymerization of norbornene with B(C_6_F_5_)_3_ as cocatalyst, and the activity reached 10^6^ g_PNB_ mol_Ni_^−1^ h^−1^. The Ni1 and Ni2 two catalysts also displayed high activities (up to 10^5^ g_polymer_ mol_Ni_^−1^ h^−1^) towards the copolymerization of norbornene and 1-hexene, and Ni2 exhibited higher activity than Ni1 did, and the catalytic activity decreased with the increased of the insertion rate of 1-hexene. The poly(NB-*co*-1-hexene)s showed better solubility and high molecular weights (up to 10^5^ g mol^−1^) than that of the PNB and were confirmed to be noncrystalline. The analyses of the structure and properties of the copolymers showed that the polymers of norbornene and 1-hexene were copolymers rather than blends of the two. In addition, the copolymerization of norbornene with 1-hexene was carried out *via* a vinyl-addition copolymerization mode and the poly(NB-*co*-1-hexene) copolymer had good thermal stability.

## Conflicts of interest

There are no conflicts to declare.

## Supplementary Material

RA-008-C8RA06561F-s001

RA-008-C8RA06561F-s002
