# Supplementary material for: Bis-(salicylaldehyde-benzhydrylimino)nickel complexes with different electron groups: crystal structure and their catalytic properties toward (co)polymerization of norbornene and 1-hexene
Source: RSC Adv. 2018 Oct 25;8(63):36298–312. doi: 10.1039/c8ra06561f (PMC9088609; doi:10.1039/c8ra06561f)
Supplement: RA-008-C8RA06561F-s001 [file RA-008-C8RA06561F-s001.pdf]

Electronic Supplementary Material (ESI) for RSC Advances.
This journal is © The Royal Society of Chemistry 2018

Bis-(salicylaldehyde-benzhydrylimino)Nickel Complexes with Different Electron Groups: Crystal Structure and Their Catalytic Properties toward (Co)polymerization of Norbornene and 1-hexene

Xiaohui He, *[a] Guangshui Tu,[a] Feng Zhang,[a] Shengmei Huang,[a,c] Changwen Cheng,[a] Chuanyi Zhu,[a] Yapeng Duan,[a] Suli Wang,[a] and Defu Chen[b]

[a]School of Materials Science and Engineering, Nanchang University, 999 Xuefu Avenue, Nanchang 330031, China
[b]School of Civil Engineering and Architecture, Nanchang University, 999 Xuefu Avenue, Nanchang 330031, China
[c]School of Materials Science and Engineering, Nanchang Hangkong University, Nanchang 330063, China
Correspondence to: X. H. He (E-mail: hexiaohui@ncu.edu.cn)

Crystallographic data for the structural analyses have been deposited with the Cambri dge Crystallographic Data Center, CCDC Nos. 1587113 for Ni1, 1575909 for Ni2, 18 17084 for Ni3, 1851039 for Ni4, 1843971 for Ni5, 1575905 for Ni6,1830324 for Ni7, 1843964 for Ni8. Copies of this information may be obtained free of charge from The Director, CCDC, 12 Union Road, Cambridge CB2 1EZ, UK (fax: +44 1223 336033; e mail: deposit@ccdc.cam.ac.ukor. http://www.ccdc.cam.ac.uk).

Synthesis of ligands (L1-L8) and nickel complexes (Ni1-Ni8)

The synthesis of the (5-CH_3)C_6H_3(OH) CHNCH(C_6H_5)$_2$ (L2) is similar to L1. L2 was got as yellow crystals. yield:1.89g(62.8%).^{1}H NMR ($CDCl_3$, δ, ppm): 16.48 (s, 1H, OH); 7.54 (s, 1H, -C=N-H);7.24-7.39(m, 10H, 2C_6H_5); 6.78-7.24 (m, 3H, C_6H_3); 5.99 (s, 1H, $C_{13}H_{12}$); 2.39(s, 3H, CH_3). FT-IR (KBr): 3044.12(w),2982.61(w),2805.91(w),

1918.79(w),1664.33(vs, $\nu_{C=N}$)1527.91(s),1464.86 (m),1416.12(w),1302.81(s).

The synthesis of the (5-OCH_3)C_6H_3(OH) CHNCH(C_6H_5)$_2$ (L3) is similar to L1. L3was got as yellow crystals. yield:1.63g(51.4%).^{1}H NMR ($CDCl_3$, δ, ppm): 12.95(s, 1H, OH); 9.42(s, 1H, -C=N-H);7.11-7.40(m, 10H, 2C_6H_5); 6.95-7.11 (m, 3H, C_6H_3); 5.62 (s, 1H, $C_{13}H_{12}$); 3.75(s, 3H, OCH_3). FT-IR (KBr): 3009.84(w), 2969.70(w), 2878.04(w), 1663.56(vs, $\nu_{C=N}$), 1528.60(s), 1468.78 (m),1417.78(w),1343.66(s).

The synthesis of the (5-Br)C_6H_3(OH) CHNCH(C_6H_5)$_2$ (L4) is similar to L1. L4 was got as yellow crystals. yield:2.31g(63.3%).^{1}H NMR ($CDCl_3$, δ, ppm): 13.54(s, 1H, OH); 9.37(s, 1H, -C=N-H);7.17-7.40(m, 10H, 2C_6H_5); 6.95-7.17 (m, 3H, C_6H_3); 5.65 (s,1H,$C_{13}H_{12}$).FT-IR(KBr):2926.85(w),2860.49(w),2755.04(w),1909.97(w),1647.73(vs, $\nu_{C=N}$), 1515.71(s),1410.03 (m),1305.76.12(s).

The synthesis of the (3-CH_3)C_6H_3(OH) CHNCH(C_6H_5)$_2$ (L5) is similar to L1. L5was got as yellow crystals. yield:1.82g(60.5%).^{1}H NMR ($CDCl_3$, δ, ppm): 13.73(s, 1H, OH); 9.49(s, 1H, -C=N-H);7.12-7.40(m, 10H, 2C_6H_5); 6.85-7.11 (m, 3H, C_6H_3); 5.61 (s, 1H, $C_{13}H_{12}$);2.31 (s, 3H, CH_3). FT-IR (KBr):2929.73(w), 2756.36 (w), 1924.44(w),1661.48(vs, $\nu_{C=N}$), 1510.84 (s),1472.50 (m),1394.00 (w),1291.95 (m).

The synthesis of the (3-OCH_3)C_6H_3(OH) CHNCH(C_6H_5)$_2$ (L6) is similar to L1. L6 was got as yellow crystals. yield:1.72 g(54.3%).^{1}H NMR ($CDCl_3$, δ, ppm): 14.16(s, 1H, OH); 9.50(s, 1H, -C=N-H);7.02-7.44(m, 10H, 2C_6H_5); 6.78-7.02 (m, 3H, C_6H_3); 5.62 (s, 1H, $C_{13}H_{12}$); 3.91(s, 3H, OCH_3). FT-IR (KBr): 3133.34(w), 2917.45(w), 2745.53(w), 1852.54(w),1535.73(vs, $\nu_{C=N}$), 1358.61 (w),1305.80 (m).

The synthesis of the(3-Br) (5-Br)C_6H_2(OH) CHNCH(C_6H_5)$_2$ (L7) is similar to L1. L7 was got as yellow crystals. yield:2.85g(64.0%).^{1}H NMR ($CDCl_3$, δ, ppm): 14.64 (s, 1H, OH); 9.32(s, 1H, -C=N-H);7.27-7.43(m, 10H, 2C_6H_5); 7.10-7.27 (m, 3H, C_6H_2); 5.69 (s, 1H, $C_{13}H_{12}$). FT-IR (KBr): 2934.87(w), 2857.52 (w), 2718.63 (w), 1917.09(w),1655.54(vs, $\nu_{C=N}$), 1607.85 (w),1512.03 (m),1474.99 (w),1309.43(s).

The synthesis of the(3-Cl) (5-Cl)C_6H_2(OH) CHNCH(C_6H_5)$_2$ (L8) is similar to L1. L8

was got as yellow crystals. yield:2.17g(61.0%).^{1}H NMR ($CDCl_3$, δ, ppm): 14.68 (s, 1H, OH); 9.30(s, 1H, -C=N-H);7.21-7.41(m, 10H, $2C_6H_5$); 7.08-7.21 (m, 3H, C_6H_2); 5.67 (s, 1H, $C_{13}H_{12}$). FT-IR (KBr): 2927.21(w), 2863.47(w), 2803.96(w), 2036.27(w),1651.19(vs, $\nu_{C=N}$), 1580.71(m),1512.42 (s),1397.37(w),1310.33(m).

The synthesis of the Ni{(5-CH_3)C_6H_3(O)CHNCH(C_6H_5)$_2$ (Ni2) is similar to Ni1. The dark-green crystal Ni2 catalyst was crystallized from dichloromethane in 61% yield. ^{1}H NMR ($CDCl_3$, δ, ppm): 9.41(s, 2H, 2C-N-H); 1.03-1.45(m, 20H, $4C_6H_5$); 7.31-7.50(m, 6H, $2C_6H_3$); 5.61 (s, 2H, $2C_{13}H_{11}$);2.13-2.49(s, 6H, $2CH_3$). ^{13}C NMR ($CDCl_3$, δ, ppm): 165.11, 142.94, 132.92, 130.91, 129.74, 127.05, 116.94, 29.57, 20.25. Analysis calculated for Ni2: C,76.34; H,6.11; N,4.34; found: C,76.32; H,6.15; N,4.29.

The synthesis of the Ni{(5-OCH_3)C_6H_3(O)CHNCH(C_6H_5)$_2$ (Ni3) is similar to Ni1. The dark-green crystal Ni3 catalyst was crystallized from dichloromethane in 58% yield. ^{1}H NMR ($CDCl_3$, δ, ppm): 9.42 (s, 2H, 2C-N-H); 0.65-1.03 (m, 20H, $4C_6H_5$); 7.31-7.49(m, 6H, $2C_6H_3$); 5.57 (s, 2H, $2C_{13}H_{11}$), 3.43-3.91(s, 6H, $2OCH_3$). ^{13}C NMR ($CDCl_3$, δ, ppm): 166.31, 143.87, 133.23, 131.31, 129.82, 128.02, 117.32, 56.32, 30.32. Analysis calculated for Ni3: C,72.90; H,5.83; N,3.95; found: C,72.84; H,5.75; N,3.97.

The synthesis of the Ni{(5-Br)C_6H_3(O)CHNCH(C_6H_5)$_2$ (Ni4) is similar to Ni1. The dark-green crystal Ni4 catalyst was crystallized from dichloromethane in 71% yield. ^{1}H NMR ($CDCl_3$, δ, ppm): 9.41 (s, 2H, 2C-N-H); 1.01-1.78 (m, 20H, $4C_6H_5$); 7.32-7.56(m, 4H, $2C_6H_2$); 5.56 (s, 2H, $2C_{13}H_{11}$). ^{13}C NMR ($CDCl_3$, δ, ppm): 164.49, 147.91, 132.92, 129.48, 129.74, 127.07, 124.23, 104.16, 29.26. Analysis calculated for Ni4: C,61.08; H,4.38; N,3.47; found: C61.08; H,4.39; N,3.46.

The synthesis of the Ni{(3-CH_3)C_6H_3(O)CHNCH(C_6H_5)$_2$ (Ni5) is similar to Ni1. The dark-green crystal Ni5 catalyst was crystallized from dichloromethane in 64% yield. ^{1}H NMR ($CDCl_3$, δ, ppm): 8.98(s, 2H, 2C-N-H); 0.75-1.67(m, 20H, $4C_6H_5$); 5.60-6.93(m, 6H, $2C_6H_3$); 5.90(s, 2H, $2C_{13}H_{11}$), 2.02-2.55(s, 6H, $2CH_3$). ^{13}C NMR ($CDCl_3$, δ, ppm): 196.67, 159.53, 134.64, 129.53, 128.76, 127.03, 118.35, 49.41,

17.23. Analysis calculated for Ni5: C,76.34; H,6.11; N,4.14; found: C,76.34; H,6.12; N,4.11.

The synthesis of the Ni{(3-OCH_3)C_6H_3(O)CHNCH(C_6H_5)$_2$ (Ni6) is similar to Ni1. The dark-green crystal Ni6 catalyst was crystallized from dichloromethane in 69% yield. ^{1}H NMR (CDCl3, δ, ppm): 9.65 (s, 2H, 2C-N-H); 0.97-1.32 (m, 20H, 4C6H5); 7.42-7.53(m, 6H, 2C6H3); 5.72 (s, 2H, 2C13H11), 3.53-3.62 (s, 6H, 2OCH3). ^{13}C NMR ($CDCl_3$, δ, ppm): 165.46, 155.09, 140.22, 133.23, 129.71, 127.73, 119.73, 56.71, 29.96. Analysis calculated for Ni6: C,72.90; H,5.83; N,3.95; found: C,72.92; H,5.84; N,3.91.

The synthesis of the Ni{(3-Br)(5-Br)C_6H_2(O)CHNCH(C_6H_5)$_2$}$_2$ (Ni7) is similar to Ni1. The dark-green crystal Ni7 catalyst was crystallized from dichloromethane in 75% yield. ^{1}H NMR ($CDCl_3$, δ, ppm): 9.33 (s, 2H, 2C-N-H); 1.35-2.41 (m, 20H, 4C_6H_5); 7.48-7.92(m, 4H, 2C_6H_2); 5.67 (s, 2H, 2$C_{13}H_{11}$). ^{13}C NMR ($CDCl_3$, δ, ppm): 163.34, 143.21, 131.82, 129.28, 128.45, 126.37, 117.72, 113.34, 31.31. Analysis calculated for Ni7: C,51.08; H,3.45; N,2.91; found: C,51.07; H,3.47; N,2.89.

The synthesis of the Ni{(3-Cl)(5-Cl)C_6H_2(O)CHNCH(C_6H_5)$_2$}$_2$ (Ni8) is similar to Ni1. The dark-green crystal Ni8 catalyst was crystallized from dichloromethane in 73% yield. ^{1}H NMR ($CDCl_3$, δ, ppm): 9.39 (s, 2H, 2C-N-H); 0.51-1.02 (m, 20H, 4C_6H_5); 7.33-7.64(m, 4H, 2C_6H_2); 5.71 (s, 2H, 2$C_{13}H_{11}$). ^{13}C NMR ($CDCl_3$, δ, ppm): 160.13, 143.18, 129.16, 128.42, 127.67, 126.07, 122.24, 120.12, 29.89. Analysis calculated for Ni8: C,62.63; H,4.23; N,3.56; found: C,62.65; H,4.25; N,3.54.

Table S1 Selected bond lengths (Å) and angles (deg) for Ni1-Ni4

**Ni1**		**Ni2**		**Ni3**		**Ni4**	
**Bond**	**Length**	**Bond**	**Length**	**Bond**	**Length**	**Bond**	**Length**
N(1)-Ni(1)	1.927(4)	N(1)-Ni(1)	1.9300(13)	N(1)-Ni(1)	1.916(2)	N(1)-Ni(1)	1.9261(16)
N(2)-Ni(1)	1.930(4)	N(2)-Ni(1)	1.9300(13)	N(2)-Ni(1)	1.916(2)	N(2)-Ni(1)	1.9261(16)
Ni(1)-O(2)	1.816(4)	Ni(1)-O(2)	1.8295(12)	Ni(1)-O(2)	1.831(2)	Ni(1)-O(2)	1.8480(14)
Ni(1)-O(1)	1.821(4)	Ni(1)-O(1)	1.8295(12)	Ni(1)-O(1)	1.831(2)	Ni(1)-O(1)	1.8480(14)
**Bond**	**Angle**	**Bond**	**Angle**	**Bond**	**Angle**	**Bond**	**Angle**

O(2)-Ni(1)-O(1)	170.83(16)	O(2)-Ni(1)-O(1)	180.0	O(2)-Ni(1)-O(1)	180.00(1)	O(2)-Ni(1)-O(1)	180.00(9)
O(2)-Ni(1)-N(1)	87.33(15)	O(2)-Ni(1)-N(1)	87.69(5)	O(2)-Ni(1)-N(1)	88.47(8)	O(2)-Ni(1)-N(1)	88.33(7)
O(1)-Ni(1)-N(1)	91.86 (15)	O(1)-Ni(1)-N(1)	92.31 (5)	O(1)-Ni(1)-N(1)	91.53 (8)	O(1)-Ni(1)-N(1)	91.67(7)
O(2)-Ni(1)-N(2)	93.55 (15)	O(2)-Ni(1)-N(2)	92.32 (5)	O(2)-Ni(1)-N(2)	91.53 (8)	O(2)-Ni(1)-N(2)	91.67(7)
O(1)-Ni(1)-N(2)	172.24(14)	O(1)-Ni(1)-N(2)	87.69(5)	O(1)-Ni(1)-N(2)	88.47(8)	O(1)-Ni(1)-N(2)	88.33(7)
N(1)-Ni(1)-N(2)	130.5 (3)	N(1)-Ni(1)-N(2)	180.0	N(1)-Ni(1)-N(2)	180.00(1)	N(1)-Ni(1)-N(2)	180.00(8)

Table S2 Selected bond lengths (Å) and angles (deg) for Ni5-Ni8

**Ni5**		**Ni6**		**Ni7**		**Ni8**	
**Bond**	**Length**	**Bond**	**Length**	**Bond**	**Length**	**Bond**	**Length**
N(1)-Ni(1)	1.921(7)	N(1)-Ni(1)	1.919(2)	N(1)-Ni(1)	1.899(3)	N(1)-Ni(1)	1.931(3)
N(2)-Ni(1)	1.921(7)	N(2)-Ni(1)	1.919(2)	N(2)-Ni(1)	1.899(3)	N(2)-Ni(1)	1.931(3)
Ni(1)-O(2)	1.826(8)	Ni(1)-O(2)	1.8298(16)	Ni(1)-O(2)	1.840(2)	Ni(1)-O(2)	1.846(3)
Ni(1)-O(1)	1.826(8)	Ni(1)-O(1)	1.8298(16)	Ni(1)-O(1)	1.840(2)	Ni(1)-O(1)	1.846(3)
**Bond**	**Angle**	**Bond**	**Angle**	**Bond**	**Angle**	**Bond**	**Angle**
O(2)-Ni(1)-O(1)	180.000(1)	O(2)-Ni(1)-O(1)	180.00(10)	O(2)-Ni(1)-O(1)	170.30(15)	O(2)-Ni(1)-O(1)	180.00(18)
O(2)-Ni(1)-N(1)	87.9(3)	O(2)-Ni(1)-N(1)	87.78(7)	O(2)-Ni(1)-N(1)	87.47(10)	O(2)-Ni(1)-N(1)	88.31(13)
O(1)-Ni(1)-N(1)	92.1(3)	O(1)-Ni(1)-N(1)	92.22(7)	O(1)-Ni(1)-N(1)	92.81(10)	O(1)-Ni(1)-N(1)	91.69(13)
O(2)-Ni(1)-N(2)	92.1(3)	O(2)-Ni(1)-N(2)	92.22(7)	O(2)-Ni(1)-N(2)	92.81(11)	O(2)-Ni(1)-N(2)	91.69(13)
O(1)-Ni(1)-	87.9(3)	O(1)-	87.78(7)	O(1)-	87.47(10)	O(1)-	88.31(13)

N(2)		Ni(1)-N(2)		Ni(1)-N(2)		Ni(1)-N(2)	
N(1)-Ni(1)-N(2)	180.000(1)	N(1)-Ni(1)-N(2)	180.0	N(1)-Ni(1)-N(2)	176.68(18)	N(1)-Ni(1)-N(2)	180.000(1)
